# A YOLOv11-based AI system for keypoint detection of auricular acupuncture points in traditional Chinese medicine

**DOI:** 10.3389/fphys.2025.1629238

**Published:** 2025-07-10

**Authors:** Ganhong Wang, Limei Yin, Hanyue Zhang, Kaijian Xia, Yue Su, Jian Chen

**Affiliations:** ^1^ Department of Gastroenterology, Changshu Hospital Affiliated to Nanjing University of Chinese Medicine, Suzhou, China; ^2^ Department of Nursing, Changshu Hospital Affiliated to Nanjing University of Chinese Medicine, Suzhou, China; ^3^ Center of Intelligent Medical Technology Research, Changshu Hospital Affiliated to Soochow University, Suzhou, China; ^4^ Department of Gastroenterology, Changshu Hospital Affiliated to Soochow University, Suzhou, China

**Keywords:** auricular acupoints, deep learning, keypoint detection, YOLO, artificial intelligence

## Abstract

**Objective:**

This study aims to develop an artificial intelligence model and web-based application for the automatic detection of 21 commonly used auricular acupoints based on the YOLOv11 neural network.

**Methods:**

A total of 660 human ear images were collected from three medical centers. The LabelMe annotation tool was used to label the images with bounding boxes and key points, which were then converted into a format compatible with the YOLO model. Using this dataset, transfer learning and fine-tuning were performed on different-sized versions of the YOLOv11 neural network. The model performance was evaluated on validation and test sets, considering metrics such as mean average precision (mAP) under different thresholds, recall, and detection speed. The best-performing model was subsequently deployed as a web application using the Streamlit library in the Python environment.

**Results:**

Five versions of the YOLOv11 keypoint detection model were developed, namely YOLOv11n, YOLOv11s, YOLOv11m, YOLOv11l, and YOLOv11x. Among them, YOLOv11x achieved the highest performance in the validation set with a precision of 0.991, recall of 0.976, mAP^50^ of 0.983, and mAP^50–95^ of 0.625, though it exhibited the longest inference delay (19 ms/img). On the external test set, YOLOv11x achieved an ear recognition accuracy of 0.996, sensitivity of 0.996, and an F1-score of 0.998. For auricular acupoint localization, the model achieved an mAP^50^ of 0.982, precision of 0.975, and recall of 0.976. The model has been successfully deployed as a web application, accessible on both mobile and desktop platforms to accommodate diverse user needs.

**Conclusion:**

The YoloEar21 web application, developed based on YOLOv11x and Streamlit, demonstrates superior recognition performance and user-friendly accessibility. Capable of providing automatic identification of 21 commonly used auricular acupoints across various scenarios for diverse users, it exhibits promising potential for clinical applications.

## Introduction

Auricular therapy, as an integral component of ancient Chinese acupuncture, has been proven to be a simple yet effective approach for disease prevention, diagnosis, and treatment (Yang et al., 2020; Liu et al., 2021). Specific points on the auricle are believed to reflect the physiological functions and pathological changes of the human body, collectively referred to as auricular acupoints. Traditional Chinese medicine (TCM) emphasizes a diagnostic approach known as “inspection, listening and smelling, inquiry, and palpation,” among which auricular identification plays a crucial role in visual diagnosis. This process primarily involves the precise localization of auricular acupoint key features. Despite its significant clinical value, mastering auricular therapy requires systematic training, extensive clinical practice, and substantial medical expertise. Moreover, individual variations in auricular morphology mean that acupoint localization cannot solely rely on standard templates, as doing so may lead to inaccurate positioning and, consequently, suboptimal therapeutic outcomes. This inherent variability presents a challenge for the clinical application of auricular therapy (Wirz-Ridolfi, 2019).

Artificial intelligence (AI), a cutting-edge technology built upon the rapid advancements in the internet and computing industries, has demonstrated immense potential across various medical fields, including assisted capsule endoscopy interpretation and colonoscopy quality control (Chen et al., 2024a; Chen et al., 2024b). The application of AI in auricular acupoint identification has shown remarkable adaptability through keypoint detection models. Since the introduction of YOLOv1 in 2015, this single-stage object detection network has undergone continuous improvements (Terven et al., 2023), incorporating algorithmic enhancements and new functionalities to achieve both higher real-time processing speeds and improved detection accuracy. In September 2024, the Ultralytics team released the latest YOLOv11 model, integrating novel structures such as the C3k2 module, SPPF, and C2PSA to enhance feature extraction and detection capabilities.

In this study, a dedicated dataset was constructed using auricular images collected from multiple centers. A deep learning-based keypoint detection model was trained and tested using the YOLOv11 neural network, and subsequently developed into a web application. This model enables the rapid localization and identification of 21 commonly used auricular acupoints, significantly improving the efficiency and accuracy of acupoint recognition, thereby facilitating the broader adoption and therapeutic efficacy of auricular therapy.

## Methods

### Datasets

**FIGURE 1:**
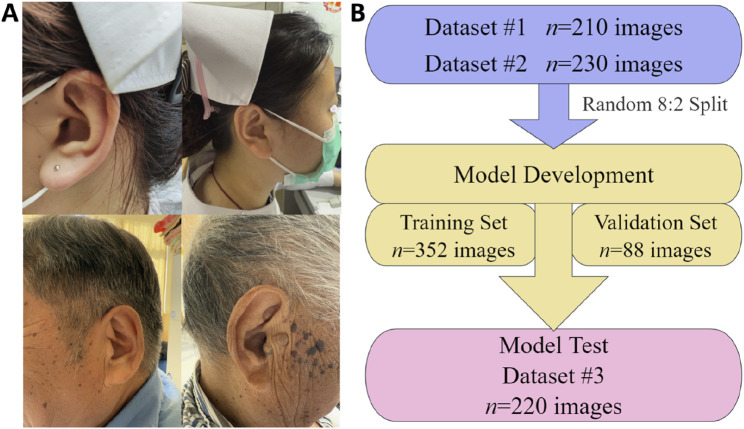
Illustrative samples and distribution of dataset images for the AI model of automatic auricular acupoint keypoint detection. **(A)** Representative images from the dataset; **(B)** distribution of image quantities across the training, validation, and test sets.

In this study, three datasets were collected, covering the period from May 2018 to December 2024. Dataset 1# (n = 210 images), Dataset 2# (n = 230 images), and Dataset 3# (n = 220 images) were sourced from the Ear210 public dataset (https://www.kaggle.com/datasets/chg0901/ear210-dataset-coco), Changshu Hospital Affiliated to Nanjing University of Chinese Medicine, and Changshu Hospital Affiliated to Soochow University, respectively. In total, 660 human ear images were gathered for model training, validation, and testing. All images underwent anonymization, with identifying metadata such as location, time, and device information removed. Additionally, the eye regions of subjects were pixelated to ensure privacy protection. Figure 1A presents representative image samples from the dataset, while Figure 1B illustrates the distribution of images across the training, validation, and test sets.

### Image annotation

**FIGURE 2:**
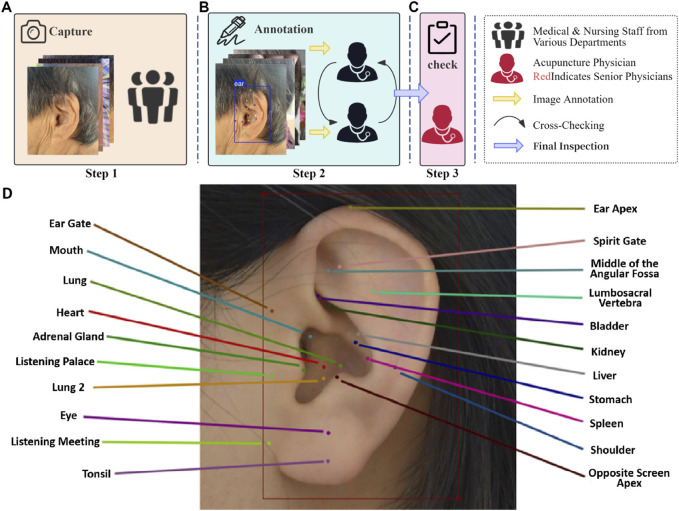
Annotation process and representative examples for the AI model of automatic auricular acupoint keypoint detection. **(A)** Step 1: Medical staff from various hospital departments collect ear images from individuals of different ages, genders, and occupations using different devices. **(B)** Step 2: Two acupuncture specialists annotate the collected images using the LabelMe 5.3.1 graphical annotation tool and perform cross-checking. **(C)** Step 3: A senior acupuncture specialist with 15 years of experience reviews the annotations and makes the final decision on the labeling. **(D)** Example of Auricular Acupoint Image Annotation. The training and validation sets were annotated by one group of acupuncture specialists, while the external test set was independently annotated by a separate group of clinicians.

The annotation of acupoints was conducted in accordance with China’s national standard *Nomenclature and Location of Meridian Points* (GB/T 12346-2021). The annotation process and representative examples are illustrated in Figure 2. In this study, the annotation workflow was divided into three stages, with participants assigned to three distinct teams, each responsible for a specific phase of the process. Only images that underwent this structured annotation and verification procedure were deemed eligible for training the deep learning model. The LabelMe 5.3.1 annotation tool (Russell et al., 2008) was utilized to label auricular images with bounding boxes and 21 auricular acupoint keypoints. The JSON files generated in the LabelMe format were subsequently converted into TXT format compatible with YOLO model training, ensuring seamless integration with the deep learning model’s requirements. Inter-rater agreement among annotators was assessed using Cohen’s kappa statistic to ensure the reliability of the annotation process.

### Image preprocessing

A diverse range of devices was used for image acquisition, including one Canon EOS R6Ⅱ camera, one iPhone 15, and two Huawei Mate 60 smartphones. To enhance the generalization capability of the trained model, a series of image preprocessing and data augmentation strategies were implemented. During preprocessing, all images were uniformly resized to 640 × 640 pixels while maintaining their original aspect ratios. In the data augmentation phase, multiple random transformation strategies were applied to simulate real-world variations in image conditions. Specifically, random horizontal flipping was applied with a 50% probability to improve the model’s robustness to mirrored transformations. Additionally, to address the potential imbalance caused by a preference for single-sided ear image collection, mirroring transformations were employed to generate artificial samples of opposite ear sides, effectively mitigating dataset bias. Furthermore, RandomResize and RandomCrop algorithms were utilized to randomly adjust image sizes and perform random cropping, allowing the model to learn multi-scale and localized features. The HSVRandomAug algorithm, provided by YOLO (Qiu et al., 2023), was employed to introduce random perturbations in the HSV color space, enhancing the model’s resilience to variations in lighting and color differences. All data augmentation operations were integrated into the training process online (Athalye and Arnaout, 2023; Kang et al., 2019), eliminating the need for pre-generated augmented images while ensuring that the model encounters subtly altered images in each iteration, thereby improving its adaptability and robustness.

### Model training configuration

**FIGURE 3:**
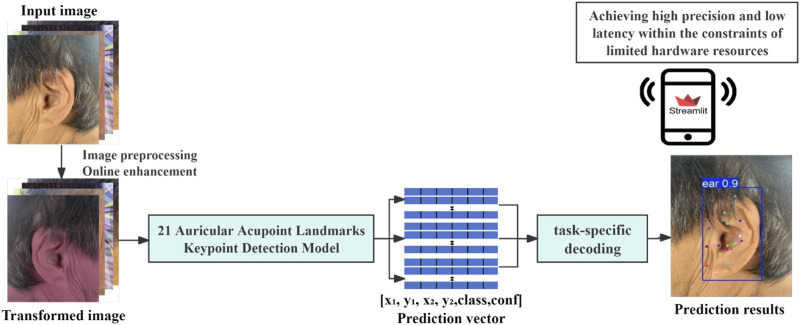
Keypoint detection system for the automatic recognition of 21 common auricular acupoints.

This study employed a transfer learning strategy (Shin et al., 2016), utilizing five different scales of YOLOv11 pose models pretrained on the COCO (Common Objects in Context) dataset: nano (n), small (s), medium (m), large (l), and extra (x). These variations represent different model sizes and complexities. During training, the model weights were randomly initialized, and all layers were retrained using the dedicated dataset developed in this study, which includes 21 commonly used auricular acupoints. The optimizer was automatically selected, and the learning rate was adjusted based on configuration settings to optimize training performance. The training process was set to a maximum of 120 epochs, with a batch size of 16. The intersection over union (IoU) threshold for performance evaluation was set to 0.6, and the maximum number of detected targets was limited to 20. To enhance efficiency, automatic mixed-precision training was enabled on the graphics processing unit (GPU). Additionally, an early stopping mechanism was implemented, with a patience setting of 8, meaning training would be terminated early if no improvement in validation performance was observed over eight consecutive epochs, thereby preventing overfitting. The proposed auricular acupoint keypoint detection system developed in this study is illustrated in Figure 3.

### Model performance evaluation

This study conducted a comprehensive evaluation of the trained model, including internal validation (n = 88) and external testing (n = 220). The performance assessment was performed using both bounding box (Box, B) and keypoint (Keypoint, K) metrics. The bounding box metrics were employed to evaluate the model’s accuracy in ear localization, while the keypoint metrics were utilized to assess the precision of keypoint detection. The evaluation metrics comprised: mean average precision for bounding boxes at IoU threshold 0.50 [mAP^50^(B)], mean average precision for keypoints at IoU threshold 0.50 [mAP^50^(K)], mean average precision for bounding boxes across IoU thresholds from 0.50 to 0.95 [mAP^50–95^(B)], mean average precision for keypoints across IoU thresholds from 0.50 to 0.95 [mAP^50–95^(K)], precision for bounding boxes [precision(B)], precision for keypoints [precision(K)], recall for bounding boxes [recall(B)], recall for keypoints [recall(K)], and latency (ms/img). The evaluation metrics were calculated according to the following definitions: Precision (Equation 1), recall (also known as sensitivity, Equation 2), mean average precision at an IoU threshold of 0.50 (mAP50, Equation 3), mean intersection over union (MIoU, Equation 4), and latency (Equation 5).

$$\text{Precision}=\frac{\text{TP}}{\text{TP}+\text{FP}}$$ (1)

$$\text{Recall}=\text{Sensitivity}=\frac{\text{TP}}{\text{TP}+\text{FN}}$$ (2)

$${\text{mAP}}^{50}=\frac{1}{N}\sum_{i=1}^{N}{\text{AP}}_{i}$$ (3)

$$\text{MIoU}\left(y,p\right)=\frac{\left|y\cap p\right|}{\left|y\cup p\right|}\times 100\%$$ (4)

$$\text{Latency}\;\left(\text{ms}/\text{img}\right)=\text{Preprocess}\;\text{Time}\;+\;\text{Inference}\;\text{Time}+\text{Postprocess}\;\text{Time}$$ (5)

True positives (TP) represent the number of correctly detected ear regions, while false positives (FP) indicate the number of instances where the model incorrectly detected a region as an ear. False negatives (FN) refer to the number of ear regions that the model failed to detect. In the equations, *y* represents the ground truth ear region, and *p* denotes the predicted ear region. The term |*y*∩*p*| refers to the number of pixels in the intersection (overlapping area) between the ground truth and the predicted region, whereas |*y*∪*p*| represents the number of pixels in the union (total covered area) of the ground truth and the predicted region.

### Web application development

To facilitate the convenient application of the auricular acupoint recognition model across different settings, such as hospitals and homes, while catering to diverse user groups including doctors, nurses, patients, and students, this study developed a web application named “YoloEar21”in the Python environment. The application was built using the best-performing model and the Streamlit library. The interface design integrates Streamlit’s native UI components with Ant Design aesthetics, creating a user-friendly and visually intuitive experience. Users can upload images and videos via the sidebar or utilize their device’s WebCam (e.g., smartphone or computer) for real-time capture. By clicking the “Detect Keypoints” button, the system automatically performs acupoint detection and inference. This application is cross-platform compatible, easy to operate, and shareable, allowing seamless functionality on both mobile and desktop devices.

**FIGURE 4:**
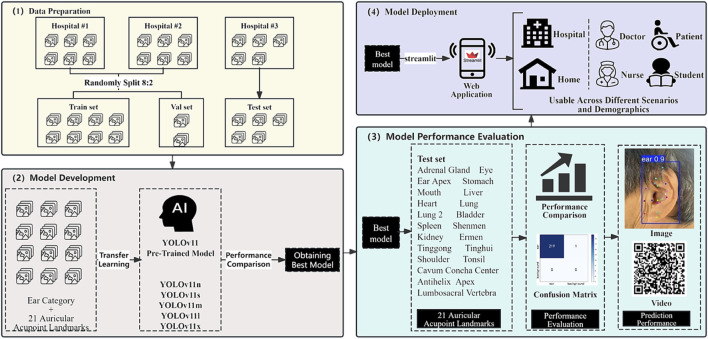
Flowchart of the AI model for automatic detection of auricular acupoint keypoints.

The workflow of this study (Figure 4) comprises four distinct phases: (1) Data Preparation: involving data collection and partitioning into training, validation, and test sets; (2) Model Development: employing transfer learning across five YOLO architectures to identify the optimal model; (3) Model Performance Evaluation: assessing the selected model through comprehensive metrics including precision, recall, and inference speed using the test set; and (4) Model Deployment: implementing the optimal model into a Streamlit-based web application designed for versatile multi-scenario applications.

### Experimental environment

The computing platform used in this study is configured as follows: an NVIDIA RTX A4000 GPU with 16.9 GB VRAM, an Intel Xeon E5-2680 v4 six-core CPU, 30.1 GB RAM, and 451.0 GB of storage. The development, training, and image processing of the deep learning model were conducted using PyTorch 1.10.1 + cu113, along with other supporting Python libraries. The training process was tracked and managed using Weights & Biases (wandb). For data processing, analysis, and visualization, the study utilized Pandas 1.3.4, NumPy 1.21.4, Matplotlib 3.5.0, and Plotly 5.4.0. Model saving and loading were handled using H5py 3.6.0. The web application was developed with Streamlit 1.36.0, and the model development environment was based on Ultralytics YOLOv8.0.145, running on Python 3.9.

## Results

### Baseline data

A total of 660 human ear images were included in this study, with baseline characteristics summarized in Table 1. The dataset comprised 316 images from male subjects (47.9%) and 344 from female subjects (52.1%). There were 323 images of left ears (48.9%) and 337 images of right ears (51.1%). In total, 660 bounding boxes and 13,860 auricular acupoint keypoints were annotated. The model development set consisted of images from Dataset 1# and Dataset 2#, totaling 440 images. These were randomly divided into a training set (n = 352) and a validation set (n = 88) using an 8:2 sampling ratio. The test set (n = 220) was sourced from Dataset 3#.

**TABLE 1:** Baseline demographic characteristics of the study dataset.

Characteristic	Training Set (n = 352)	Validation Set (n = 88)	Test Set (n = 220)	Total (n = 660)
Sex (Male/Female)	171/181	45/43	100/120	316/344
Ear side (Left/Right)	173/179	45/43	105/115	323/337
Age (years)	69.32 ± 13.33	71.24 ± 12.51	70.53 ± 13.72	69.95 ± 13.35
Ethnicity (Han/Others)	348/4	85/3	215/5	648/12

Note: “Han” refers to the Han Chinese, the largest ethnic group in China. Age is presented as mean ± standard deviation (SD). Disease status was not restricted or collected, as this study focused on general auricular acupoint localization in a broad population.

### Model training

**FIGURE 5:**
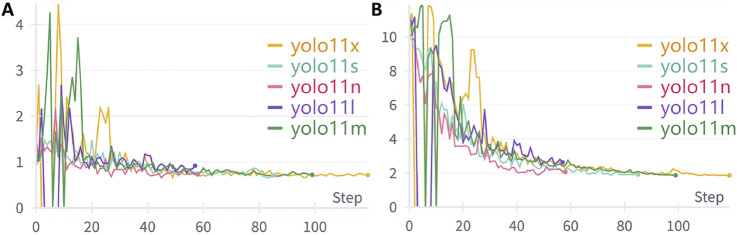
The variation trends of loss functions during the training process of different YOLOv11 model versions. **(A)** The variation trend of Bounding Box Loss across training epochs; **(B)** The variation trend of Keypoint Loss across training epochs.

The study utilized wandb to track the complete training process of the model. Figure 5 illustrates the loss function trends of different YOLOv11 model versions during the training phase. The displayed losses include bounding box loss, which measures the accuracy of ear localization, and keypoint loss, which evaluates the precision of auricular acupoint detection. As the number of training epochs increased, the model’s loss values gradually decreased and stabilized, indicating that the model was converging towards an optimized state.

**FIGURE 6:**
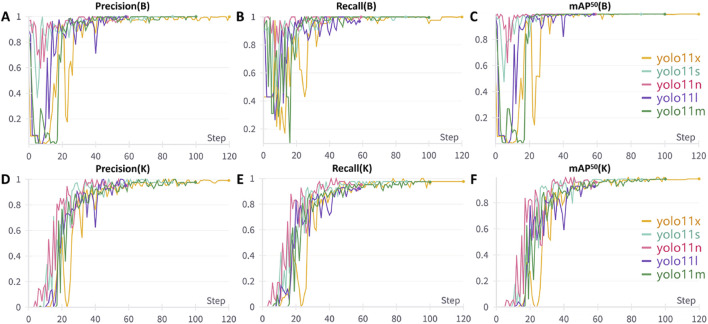
Performance Metric Trends of Different YOLO Models During Training. **(A)** Variation trend of bounding box precision; **(B)** Variation trend of bounding box recall; **(C)** Variation trend of bounding box mAP50; **(D)** Variation trend of keypoint precision; **(E)** Variation trend of keypoint recall; **(F)** Variation trend of keypoint mAP50. B: Bounding Box, K: Keypoint, mAP^50^: mean average precision at 50% Intersection over Union threshold.

Figures 6A–C illustrate the variations in precision, recall, and mAP50 performance metrics for ear bounding box localization across different models throughout the training process. Initially, these metrics exhibit a slow increase with significant fluctuations but gradually stabilize and maintain high values upon convergence. Notably, all five model versions demonstrated minimal differences in ear bounding box localization performance, each achieving an optimal level exceeding 0.995. Figures 6D-F and F depict the trends in precision, recall, and mAP^50^ for the localization of 21 commonly used auricular acupoint keypoints during training. In the early stages, the performance metrics showed fluctuations while improving progressively, eventually stabilizing at their peak values. Among the models, YOLOv11x exhibited the best overall performance across all keypoint detection metrics, achieving a precision of 0.991, recall of 0.976, and mAP^50^ of 0.983.

### Model performance evaluation

**FIGURE 7:**
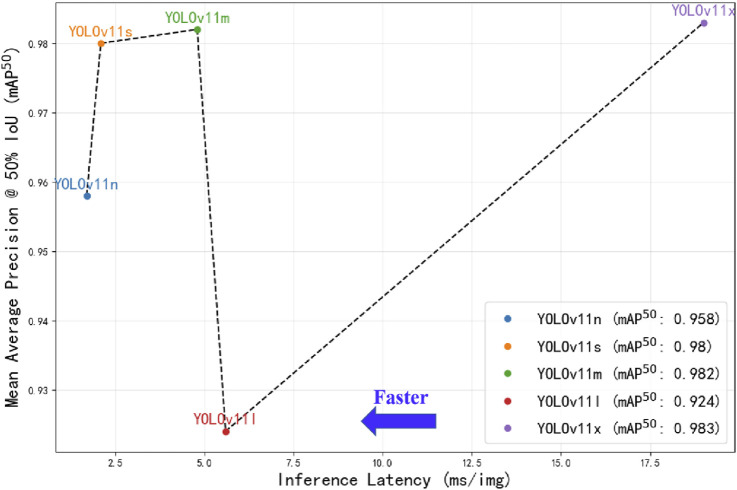
Performance comparison of different YOLOv11 model versions. The x-axis represents the inference time required for processing a single image by different YOLOv11 models under the PyTorch framework, measured in milliseconds (ms), where positions further to the left indicate faster processing speeds; the y-axis displays the mean average precision at 50% Intersection over Union threshold (mAP50) obtained by the models on the validation set, with higher positions indicating greater mAP50 values.

A comparative analysis of different versions of the YOLOv11 model was conducted on the validation set (Table 2). The results showed that YOLOv11x achieved the best mAP^50^ performance, although its inference speed was relatively slower. Specifically, YOLOv11x attained an mAP^50^ of 0.995 for ear localization and 0.983 for auricular acupoint recognition, with an average inference time of 19 milliseconds per image (equivalent to processing 52.63 images per second). Statistical analysis indicated that the mAP^50^ for auricular acupoint recognition achieved by YOLOv11x was significantly higher than those of YOLOv11n, YOLOv11l, and YOLOv11s (p < 0.05), while there was no significant difference compared to YOLOv11m (p > 0.05). The overall trade-off between accuracy and processing speed is illustrated in Figure 7.

**TABLE 2:** Performance comparison of different YOLO versions in object detection and keypoint detection.

Model	Precision (B)	Recall (B)	mAP^50^ (B)	mAP^50–95^ (B)	Precision (K)	Recall (K)	mAP^50^ (K)	mAP^50–95^ (K)	Latency (ms/img)
yolo11n	0.997 (0.98–1.00)	1 (0.99–1.00)	0.995 (0.98–1.00)	0.825 (0.77–0.89)	0.950 (0.92–0.98)	0.952 (0.92–0.98)	0.958 (0.93–0.98)	0.536 (0.46–0.58)	1.7
yolo11s	1 (0.99–1.00)	1 (0.99–1.00)	0.995 (0.98–1.00)	0.838 (0.78–0.89)	0.976 (0.95–0.99)	0.976 (0.95–0.99)	0.980 (0.94–0.99)	0.622 (0.57–0.67)	2.1
yolo11m	1 (0.99–1.00)	0.996 (0.98–1.00)	0.995 (0.98–1.00)	0.832 (0.77–0.89)	0.976 (0.95–0.99)	0.973 (0.94–0.99)	0.982 (0.96–0.99)	0.623 (0.57–0.67)	4.8
yolo11l	1 (0.99–1.00)	0.968 (0.93–0.99)	0.994 (0.97–1.00)	0.779 (0.71–0.84)	0.951 (0.91–0.98)	0.920 (0.88–0.97)	0.924 (0.89–0.96)	0.463 (0.42–0.51)	5.6
yolo11x	0.998 (0.98–1.00)	1 (0.99–1.00)	0.995 (0.98–1.00)	0.855 (0.80–0.90)	0.991 (0.97–0.99)	0.976 (0.95–0.99)	0.983 (0.97–0.99)	0.625 (0.57–0.68)	19

Note: Performance comparison of different YOLOv11 model versions on the validation set (n = 88 images), with evaluation metrics categorized into bounding box (B) and keypoint (K) groups; latency represents the inference time required per image, measured in milliseconds (ms). mAP^50^: mean average precision at 50% Intersection over Union threshold; mAP^50–95^: mean average precision across Intersection over Union thresholds from 50% to 95%. Values in parentheses indicate 95% confidence intervals calculated by bootstrap resampling.

### Model visualization and interpretation

**FIGURE 8:**
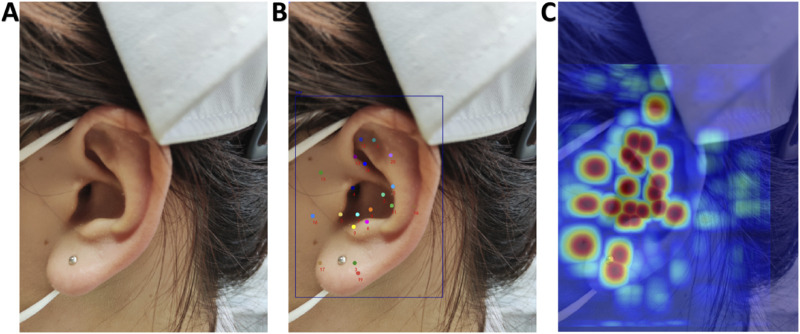
Grad-CAM Visualization of the AI Model’s Decision-Making Process. **(A)** Original images; **(B)** AI model recognition results; **(C)** Overlay display of activation heatmaps on original images.

Figure 8 demonstrates the visualization results of Grad-CAM technique in the AI model’s decision-making process. Column A displays the original ear images; Column B presents the detection outcomes from the YOLOv11x model, including both the ear object detection results and the localization of 21 auricular acupoint keypoints; Column C overlays the activation heatmaps onto the original images. In the heatmaps, warm colors (such as red and yellow) indicate regions of high model attention during decision-making, which are typically associated with critical pathological features or target characteristics, while cool colors (such as blue and purple) represent areas of lower model attention, corresponding to background or less influential regions for the model’s judgment.

### Model deployment and testing

**FIGURE 9:**
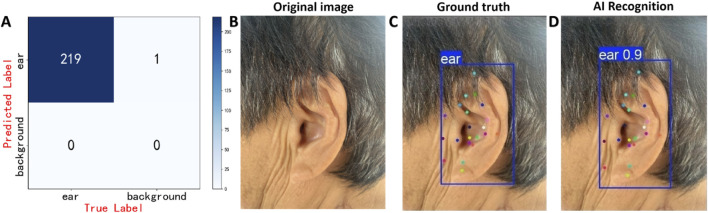
Prediction results of the model on the test set. **(A)** Confusion matrix of the AI model for ear detection task, **(B)** displays the original ear images, **(C)** presents the manually annotated images, **(D)** shows the AI model’s prediction results. The model’s predictions (Figure 9D) closely resemble the physician’s annotations (Figure 9C). In Figure 9D, the confidence score of 0.9 for the model’s prediction of the ear is displayed in the upper left corner of the predicted bounding box.

On the external test set (n = 220), YOLOv11x achieved an accuracy of 0.996, a sensitivity of 0.996, and an F1 score of 0.998 for ear detection, with the confusion matrix presented in Figure 9A. The recognition performance of 21 auricular acupoints demonstrated an mAP^50^ of 0.982, a precision of 0.975, and a recall of 0.976. Inter-rater agreement among annotators was assessed using Cohen’s kappa statistic, with a kappa value of 0.87. Additionally, a randomly selected image from the external test set was used for prediction, with its detection results presented as an example in Figures 9B–D.

**FIGURE 10:**
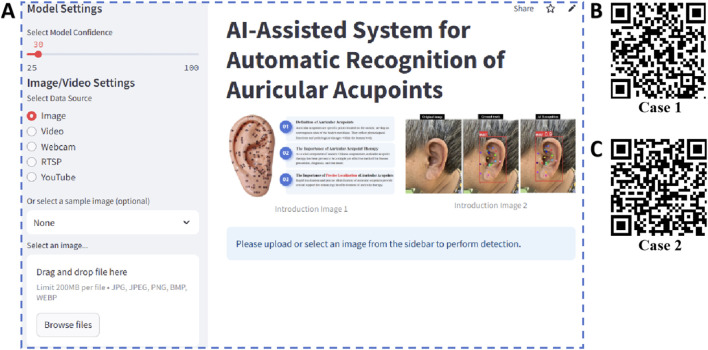
Web application developed based on the optimal model and its use cases. By scanning the QR codes in the figure, viewers can observe two real-time detection cases of the AI model performing ear recognition. **(A)** Operational interface of the web application; **(B)** Case 1; **(C)** Case 2.

The web application YoloEar21 (https://ear-spotter-app-v6.streamlit.app/), developed based on the YOLOv11x model and Streamlit library, features an operational interface as shown in Figure 10A. This application supports users in uploading ear images or videos, or utilizing the device’s camera for real-time capture. Upon clicking the prediction button, it automatically identifies the human ear and 21 auricular acupoint keypoints. Video 1 (Figure 10B) demonstrates the process of using YoloEar21 on an Apple iPhone to capture ear images via the camera and automatically detect auricular acupoint keypoints. Video 2 (Figure 10C) illustrates the real-time detection of ear images displayed on an iPad using a Logitech camera on a local computer.

## Discussion

Auricular acupoints, located on the auricle, serve as key convergence points of the body’s meridians, playing a crucial role in disease diagnosis and treatment. Modern medicine has increasingly focused on auricular acupoints and systematic auricular therapy to enhance the diagnostic and therapeutic efficacy for various conditions. Researchers worldwide have conducted numerous studies on auricular therapy, achieving remarkable clinical outcomes. For instance, auricular massage has been used to treat insomnia (Zhuang et al., 2022), auricular acupuncture has been applied to alleviate pain severity in patients with acute and chronic pain (Elliott et al., 2024), and auricular acupressure has been employed to relieve wheezing symptoms in patients with chronic obstructive pulmonary disease (COPD) (Dang et al., 2024). Due to its proven therapeutic efficacy, auricular therapy has been widely adopted across multiple medical specialties. However, its clinical application largely relies on empirical practice, lacking objective and quantifiable diagnostic criteria, which poses challenges for its standardization and broader adoption. In this study, five models were trained, among which YOLOv11x demonstrated the best performance across key metrics, including Precision, Recall, and mAP50. Although its latency is relatively high, processing at 52.63 frames per second (fps), it still meets the ISO/IEC IEEE 29119 standards for real-time performance in AI-powered medical devices (>16 fps), ensuring that clinicians receive timely and dynamic identification feedback.

Currently, research and application of auricular therapy have been conducted in dozens of countries and regions, with the World Health Organization (WHO) officially recognizing it as an effective method for treating various diseases. As modern medicine continues to integrate with traditional Chinese medicine, the application of AI technology in auricular acupoint recognition not only presents new possibilities for the inheritance and innovation of traditional medicine but also opens up vast industrial prospects and development potential, fostering an innovative “Traditional Chinese Medicine + AI” model. Li et al. (Li et al., 2023) proposed an AI model based on image segmentation algorithms, achieving auricular acupoint region localization and segmentation. This study integrates object detection and keypoint detection algorithms, enabling the developed model to first automatically identify the ear’s position in an image and then further recognize 21 commonly used auricular acupoints. The model demonstrated outstanding performance in ear localization, achieving an accuracy of 0.996 and a sensitivity of 0.996. Similarly, for auricular acupoint recognition, the model exhibited excellent performance, with an mAP50 of 0.982, a precision of 0.975, and a recall of 0.976.

As a time-honored therapy in traditional Chinese medicine, auricular therapy is not only applied in medical institutions under the guidance of healthcare professionals but is also widely practiced by patients and their families in the long-term management of chronic conditions such as insomnia and chronic pain, demonstrating its unique value. However, the accurate identification of auricular acupoints relies on specialized knowledge and training, and is influenced by factors such as the morphological variability of the auricle and anatomical differences between the bilateral ears. These complexities make it challenging for non-medical professionals to accurately locate auricular acupoints (Godson and Wardle, 2019). To address this issue, this study developed the AI model and web application YoloEar21. Through internal validation and external testing, results indicate that its efficiency and accuracy are comparable to those of experienced acupuncture specialists. More importantly, YoloEar21 features an intuitive visual interface, user-friendly operation, and strong adaptability, making it suitable for applications in hospitals, home care, and elderly care institutions. This significantly enhances the accessibility and dissemination of auricular therapy. Additionally, this AI model aligns with the traditional Chinese medicine principle of “preventing diseases before they arise,” holding great potential in early disease prevention and health management by providing precise and convenient healthcare solutions for a broader patient population. Furthermore, the system can be utilized in medical education and training, enabling medical students and practitioners to learn auricular acupoint localization more intuitively, thereby improving learning efficiency and practical proficiency.

This study has several limitations. First, the dataset is limited in size and diversity, and should be expanded to include samples from more ethnic groups and additional medical centers to improve generalizability. Second, prospective human-machine comparison studies with acupuncturists of varying experience are needed to evaluate the model’s clinical performance. Currently, YoloEar21 enables recognition of user-uploaded images on mobile and desktop platforms. In the future, we plan to expand YoloEar21 to support RTSP video streams from fixed surveillance cameras for real-time auricular acupoint detection. This would allow patients to simply sit at a designated location while the system assists healthcare professionals in real-time acupoint localization, further enhancing clinical convenience and intelligence.

## Conclusion

This study developed YoloEar21, an intelligent recognition system for 21 commonly used auricular acupoints, based on the YOLOv11 model. The system demonstrated excellent performance, achieving an mAP^50^ of 0.982 and a recall of 0.976. Through a cross-platform web application, YoloEar21 enables seamless deployment on both mobile and desktop devices, offering strong adaptability across various clinical settings. It provides real-time auricular acupoint recognition services for both physicians and patients, serving as a critical support tool for the effective application of auricular therapy.

## Data Availability

The raw data supporting the conclusions of this article will be made available by the authors, without undue reservation.
